# Institutional excellence reloaded: a 17-years, two-phase in-depth study of corporate culture change in the health care sector

**DOI:** 10.1108/JHOM-03-2020-0103

**Published:** 2021-01-27

**Authors:** Margitta B. Beil-Hildebrand

**Affiliations:** Institute of Nursing Science and Practice, Paracelsus Medical University , Salzburg, Austria

**Keywords:** Corporate culture change, Labour process analysis, Management control, Hospital management, Nursing work

## Abstract

**Purpose:**

This ethnographic revisit of a general hospital aims to critically explore and describe the mechanisms of corporate culture change and how institutional excellence is facilitated and constrained by everyday management practices between 1996/1997 and 2014/2015.

**Design/methodology/approach:**

A five-month field study of day-to-day life in the hospital's nursing division was conducted by means of an ethnographic revisit, using participant-observation, semi-structured interviews, free conversations and documentary material.

**Findings:**

Using labour process analysis with ethnographic data from a general hospital, the corporate culture is represented as faceted, complex and sophisticated, lending little support to the managerial claims that if corporate objectives are realised, they are achieved through some combination of shared values, beliefs and managerial practices. The findings tend to support the critical view in labour process writing that modern managerial initiatives lead to tightened corporate control, advanced employee subjection and extensive effort intensification. The findings demonstrate the way in which the nursing employees enthusiastically embrace many aspects of the managerial message and yet, at the same time, still remain suspicious and distance themselves from it through misbehaviour and adaptation, and, in some cases, use the rhetoric against management for their own ends.

**Practical implications:**

What are the implications for clinical and managerial practitioners? The recommendations are to (1) develop managerial practitioners who are capable of managing change combined with the professional autonomy of clinical practitioners, (2) take care to practise what you preach in clinical and managerial reality, as commitment, consent, compliance and difference of opinion are signs of a healthy corporate culture and (3) consider the implications between social structures and human actions with different work behaviours on different levels involved.

**Originality/value:**

This ethnographic revisit considers data from a labour process analysis of corporate culture change in a general hospital and revisits the ways in which contradictory expectations and pressures are experienced by nursing employees and management practitioners spread 17 years apart.

## Introduction

Throughout the late 1980s and 1990s, there was ample support for the managerial idea of mobilising corporate culture as a way of replacing rigid bureaucracies and unclear communication lines with more people-centred, decentralised, humanitarian organisations involving cultural ideologies to coordinate productive and service-oriented activities ( Peters and Waterman, 1982 ; Deal and Kennedy, 1982 ). The shift towards the novel management of corporate culture was also considered a fashionable concept in the trend of creating more humanistic and flexible healthcare organisations ( Sproat, 2001 ; Kramer and Schmalenberg, 1988 , Sullivan Havens *et al.* , 1999 ; Buchan, 1999 ). Academic promises towards the mobilisation of corporate culture were also set in relation to transformational leadership, structural empowerment and superior responsibility ( Kelly *et al.* , 2011 ; Aiken *et al.* , 2008a , b ). Yet there is a gap between the theoretical notion of how corporate culture can be changed and its actual implementation in healthcare organisations ( Ackroyd and Bolton, 1999 ; Learmonth, 2003 ; Tonkens *et al.* , 2013 ).

This ethnographic revisit investigating corporate culture change at *Jo-care* – a successful hospital in Germany – aims to understand the way a healthcare organisation functions, to assess the nature of the contemporary forms of cultural initiatives within the wider social and political context and to consider the extent to which management intends to and is effective in changing the people.

## Background

From 1996/1997 onwards, the framework of the German healthcare system changed continuously by becoming increasingly competitive, by turning from cost-containment to cost-control and cost-management initiatives ( Busse and Blümel, 2014 ). Healthcare plays a central role in the economies of most developed countries. This is certainly true in German-speaking countries where healthcare spending is usually higher than in any other European country. Overall healthcare expenditures in Germany now account for 11.2% of the gross domestic product, easily making it one of the country's largest economic sectors ( OECD, 2019 ). Despite these efforts, the healthcare sector is ailing. Although the financial crisis of 2008 was not as detrimental to the German economic structure as it was to other countries, overall healthcare services flexibility and efficiency in Germany still lag significantly behind other European countries ( Porter and Guth, 2012 ). Surprisingly, the vast majority of healthcare research has focused on clinical issues or questions of healthcare policy, but typically ignored or under-emphasised the role of healthcare organisations ( Provan *et al.* , 2004 ).

Political efforts to get more for less from the German nursing workforce date back to the healthcare reforms in the late 1990s, and the nursing profession within hospitals has clearly been subject to successive waves of cutbacks in terms of full-time-equivalents, among other cost-saving strategies. Organisational studies have found that nursing professionals display professional values, beliefs and practices as they wish to provide patient-centred care to the patient clientele. Yet, there is pressure by managerial rhetoric and practices, which force professional nurses to conform to standards of nursing care, similar to an assembly line ( Ackroyd and Bolton, 1999 ; Cooke, 2006 ; Weinberg, 2003 ).

Throughout the late 1980s and 1990s, few change management concepts have attracted as much attention as “corporate culture” within the healthcare sector. Indeed, academic journals are full of specialised articles on how institutional excellence can be facilitated through culture change initiatives by simply considering the strength of existing cultural attributes ( Hatch *et al.* , 2015 ; Saame *et al.* , 2011 ). This ongoing management and academic interest is caused by two elements: (1) the assumption that the performance of a healthcare organisation is dependent on the extent to which nursing professionals' values, beliefs and practices are aligned with management strategy, and (2) the view that the corporate culture of a healthcare organisation is subject to conscious manipulation by healthcare managers.

By the late 1990s, reviews about the mobilisation of corporate culture were not only critical of it but also raised concerns about its theoretical validity and practical utility ( Thompson and Findlay 1999 ). Others critiqued that management writers naively assume cultural unity and universality while neglecting differences across professions, sectors and organisations ( Lok *et al.* , 2011 ; Weinberg, 2003 ). Subsequently, reviews moved on to use post-structural theories which are much more sophisticated and have been much more cautious in interpreting mobilisation of corporate culture ( Casey, 1995 ; Willmott, 1993 ). In addition, critical organisational researchers refute claims that a corporate culture can be manipulated in any way as it is an integral part of an organisation, but employees are not passive to the process of corporate culture change in healthcare organisations ( Bolton, 2004 , 2005 ; Cooke, 2006 ; Muzio *et al.* , 2008 ). It is argued that nursing professionals can see through or even challenge the actions of those attempting to control their values, beliefs and practices. The creation of an iterative loop between healthcare managers, nursing professionals and the patient clientele leads to enhanced knowledge and skills; however, improved cost-effectiveness and employee participation are too often merely one end of the deal. The interest of management in managerial initiatives towards corporate culture change within the healthcare sector remains insatiate, whereas day-to-day practice is often displeasing or disappointing with unfulfilled promises and inequitable workplace relations ( Thompson, 2003 ).

The critical in-depth evaluation of corporate culture change is comparatively uncommon from an interpretative social science perspective and deserves further attention. Organisational studies on the mobilisation of corporate culture rarely investigate institutional excellence with detailed and longitudinal social, political and economic analyses. The lack of research knowledge about thorough and repeated accounts of the mobilisation of corporate culture asks for the comprehensive interpretation and description of qualitative rather than merely quantitative change. On these grounds, no single approach to the study of healthcare work has been more effective than an ethnography (i.e. *in situ* field research) in uncovering the implicit skills, decision rules, complexities, discretion and control of activities that have been labelled autonomous, skilled, ethically decisive and even essential ( Hammersley and Atkinson, 1983 ).

## Methodology

### Ethnographic revisit and the conduct of the researcher

Researchers have used the ethnographic method to understand how people carry out their respective work: the conceptual tools and the strategies employees use to accomplish their work when faced with failures, bottlenecks, increased pace, defective material and so forth ( Hatch *et al.* , 2015 ; Thursfield, 2015 ). They have also used fieldwork to study how employees are influenced by social, political and economic structures; how the same people reconcile the contradictory demands between quality and efficiency; as well as the individual and group-level processes by which they maintain dignity and control over themselves and their clientele.

This research work is organised as an “ethnographic revisit” ( Burawoy, 2003 ), returning to the site of a previous organisational study and by applying the perspective of labour process theory as the appropriate theory ( Beil-Hildebrand; 2005) . It intends to combine a critical review of the managerial claims about mobilising the corporate culture of a hospital with a second organisational ethnography. This *in situ* field research provides rich and contextualised understandings and explanations of health service work, hospital workplaces and nursing professionals and investigates systems of managerial control alongside modes of resistance, adaptation and consensus. While it is accepted that initiatives of corporate culture change can provide executive as well as professionally desired, cost-effective and innovative outcomes, as it did in the first period of this study 17 years ago ( Beil-Hildebrand; 2005) , it was critiqued that the “optimism of the management writers and others is better supported […] than the pessimism of the control school” ( Rosenthal *et al.* , 1997 , p. 497). This critique point was taken as an impetus to extend the focus of this ethnographic revisit by:

1. Highlighting the broader environmental and corporate context as well as the power resources of people inside and outside the hospital's labour process.
2. Examining the deployment of a managerial rhetoric and practices of corporate culture change to explore how the use of bureaucratic and normative control is implicated in processes of organisational change.
3. Addressing the disparity between the claims of the managerial rhetoric and the reality of its day-to-day practice and how nurses' common sense of identity and values as well as their working conditions make them responsive to corporate culture change.

### Research questions

A successive ethnographic road without seeking constancy across two field encounters while exploring and describing variations in context, in particular, to comprehend differences over time was pursued. Identical interview questions as in the first ethnographic visit in 1996/1997 were used in the interviews covering the respondents' perception of the salient features of the continuing corporate culture change initiatives that prevailed in the hospital:

1. What long-term effect has the idea of mobilising corporate culture had on nursing professionals and their healthcare work?
2. What kinds of changes have taken place in the hospital, and how have these intended and unintended changes registered in the workplace?
3. How do employees, hospital management practitioners and nurses alike experience the outcomes of continuing corporate culture initiatives?
4. What impact have the healthcare employees had upon the hospital, its management and its patient clientele?
5. What is the most crucial problem facing the selected hospital organisation within its broader economic systems, and which strategies might best resolve this problem?
6. How are workplace problems related to changes and problems in other aspects of nursing professionals' life?

### Fieldwork, data collection and analysis

The ethnographic revisit took place between November 2014 and March 2015, and the term “Jo-care's people” is used to refer to all staff, pool and charge nurses, employed by the hospital. Human subjects' own accounts and practices were used to gain insight into the hospital's history, culture and the continuing change efforts. To ensure comparability of results, the same researcher used identical methods of data collection and analytical techniques. Similar methodological procedures and data sources (participant observation, interviews, free conversations, documentary material) were applied in both study periods (1996/1997, 2014/2015).

Participant observation techniques were used in two ways: (1) the researcher was an active participant on the ward, performing duties as a nurse and observing the day-to-day practice by partaking in various meetings, seminars and so forth, and (2) the researcher observed managerial techniques by utilising the wandering around approach and other strategies used by the chief nursing executive (CNE) and his deputy. Besides observing, field notes, which were clearly dated and kept in an ongoing log in the form of a paper notebook, were taken. The notes contained brief sentences, phrases, words and sometimes quotes without the use of personal identifiers to ensure anonymity. Each day, the researcher transcribed the handwritten notes into a personal notebook computer. In addition, documentary material including publicly available healthcare reports and documents as well as a range of internal reports and communiqués was collected. Combined with observational material, these documents formed the basis for the in-depth analysis presented in this ethnography.

The data collection also involved free conversations and semi-structured interviews among organisational members from a range of differing functions and a number of different levels of responsibility. The interviewees were selected consecutively based on convenience with the aim to interview a representative proportion of employees from each professional stratum within the hospital. Each interview was conducted individually and lasted approximately 25–60 min. In total, 24 interviews were conducted using a portable digital audio-recorder, then transcribed verbatim by a research assistant and subsequently translated back and forth ( Wild *et al.* , 2005 ). Data analysis of transcripts systematically followed the labour process approach to thematic analysis to depict and discern latent trends of change management procedures.

### Limitations and reflexive ethnography

From my own experience as a previously practising nurse and CNE, I realised in the beginning of my ethnographic revisit that there was a great deal I did not and could not know. For instance, I no longer knew all of Jo-care's people and had limited knowledge of their current day-to-day practice. All the gaps in my knowledge committed me to work with them and learn more about the cultural reality in the course of this ethnographic revisit. However, I was also aware that the hospital was and still is in a state of continuous flux and change. As a result, I made it a point to start the ethnographic revisit with a clean slate, knowing so much time had passed. This highlights a particular degree of uncertainty about my fieldwork in 1996/1997 and 2014/2015, but takes into consideration my “post-fieldwork” attitude that “there is no end to this process” ( Cohen, 1992 , p. 343). Besides the hospital, its people and its processes, I have also changed since my first ethnographic visit. Considering questions of meaning, everyone changes their minds about meaning over time; that is, they do not merely change their use of particular words and actions, but also statements and identities change over time as one accumulates knowledge and experience. As a result, ethnographic interpretations can be merely regarded as temporary and are therefore continually subject to revision as time does not stand still for the informants and the researcher. In this sense, an altered understanding after 17 years was inevitable because the human subjects in the field as well as the ethnographic researcher experienced challenges and changes. In this section, I have shown the course of ethnographic reflexivity and made an attempt to address the conduct of human agency within and beyond the field.

## Findings: mechanisms, facilitators and constraints of corporate culture change

### Background to Jo-care's case

In the early 1990s came the implementation of a number of healthcare reforms in Germany, overhauling and replacing the financing of the existing healthcare system, which led to the introduction of an extensive change programme by Jo-care's ecclesiastical managers. By 1992, the hospital authority had introduced a managerial control system in the form of advanced information technology, but, more importantly, it appointed new management consultants as chief executive officers (CEO) from the outside, which set economic, political and social forms in place that were qualitatively different from those of the old Jo-care. Today, the hospital's CEO is a managerial employee of a for-profit hospital chain. In addition, the management board has grown to an unofficial six-member directorate under the supervision of Jo-care's CEO, but Jo-care itself is still politically dependent upon the Church's global aid agency.

### The official story: on the surface – then and now

The official reason for creating an extensive change programme derived from the hospital's perception of its need to respond to external and internal triggers of change. Described by the CEO and his partners as organisational development in 1996/1997, the overall programme of corporate culture change included the following elements: a service orientation within a particular workflow by implementing a patient-oriented day-to-day programme, planned exercises in normative staff re-education and a range of developmental and participatory schemes aimed at convincing Jo-care's people that there were no alternatives to the reforms, which the CEO and his partners had instigated.

In 2014/2015, however, the focus of the managerial rhetoric on the normative re-education of Jo-care's people was reduced and now focuses on the hospital's process-orientation to enhance the efficiency and quality of its healthcare services. Between 1996/1997 and 2014/2015, the conceptual and structural incorporation of values, beliefs and practices was followed through by, for example, cross-departmental conference and meeting structures on different hierarchical and organisational levels.

We also see each other at the meeting of the nursing management committee together with the nurses in charge of the departments of diagnostic and therapy where we can exchange information, […] and then we also often work in terms of content. So, whether it is on the unplanned absence concept or some procedures that we […] could change in order to achieve certain things. (charge nurse)

As in 1996/1997, the invisible normative projects provoked not just consent and compliance but also resistance and misbehaviour amongst Jo-care's people. Although these new structures and processes led to a partial relief of the nursing workload on the wards due to fewer administrative duties, the increase of the responsible autonomy of the nurses in charge of wards and the growth of the professional autonomy of the primary and associate nurses led to considerably higher workloads, and thus caused an increase in accountability towards Jo-care's CEO and his executive and senior managers.

In 1996/1997, research findings suggested that Jo-care's rhetoric was considered an important element because management and staff alike spent a considerable amount of time in attempting to introduce it into their day-to-day practice. This seemed to no longer be the case in 2014/2015 as the normative project had been implemented on a structural level, and hence lost some of its visibility. The process orientation influenced all wards and department, that is, refining the quality management due to the hospital's perpetual strive for accreditations and certifications.

### The unofficial story: below the surface – then and now

The unofficial story takes into account the various structural and procedural changes that took place between 1996/1997 and 2014/2015, and the reflexive ethnographic researcher observes the long-term impact of these changes in light of Jo-care's rhetoric. First, there is a need to address the context of labour process analysis noting the hospital's situation at the different time points characterised by continuous winds of change on very different analytical levels. Labour process theorists analyse the implementation of specific initiatives in terms of attempts at managerial control and employees' ability to resist, comply or concur within the confines of the capitalist workplace ( Thompson, 2003 ). Their epistemological and ontological view assumes a critical reality that acknowledges the possibility of people being active human subjects, who, through their mediated interplay with broader economic and institutional structures, make their own histories. The implementation of specific managerial initiatives at Jo-care had not only led to bureaucratic and normative control devices but also to an enhanced utilisation of Jo-care's people. Whereas in 1996/1997 normative control mechanisms were a main concern of the hospital's CEO and his partners, in 2014/2015, bureaucratic control mechanisms were much more emphasised and enhanced by advanced technological and normative developments reinforcing an even more sophisticated kind of managerial control, physical and mental work intensification and the attainment of people's creativity and active consent.

### Employee participation and staff development

In 2014/2015, the idea of corporate culture change no longer took centre stage, but just as in 1996/1997, the managerial rhetoric proclaimed the empowerment and the professional and individual development as the main objective of the institutionalised human resource development strategies. The resource allocation introduced in the mid-1990s carried on in 2014/2015, and it gave Jo-care's people in charge of wards and departments of diagnostic and therapy much more autonomy, but also intensified accountability.

Although the mobilisation of the corporate culture was no longer an obvious managerial topic, the strategies of normative control have been one important part of the hidden managerial agenda. Jo-care's day-to-day life with project-groups and quality circles was still carried out, but an inter-professional selection took place which excluded some of Jo-care's people. In 1996/1997 as well as 2014/2015, the managerial approach was contradictory, because, although the hospital employees did get involved regularly, they did not acquire the ability to display more independence.

And that really dawned on me, this is just a minor example, at the planning board. At the time, a lot of people sat down and did a lot of thinking about how a planning board should look like and then at some time it was suddenly there. Just like it should have looked right from the beginning. And then I thought to myself, this is all just a scam. (charge nurse)

The manipulation of the context of nursing work without questioning the professional autonomy of nurses can be seen as one key element of the overwhelming success of Jo-care's rhetoric. In 2014/2015, the flexibility within the day-to-day practice of the hospital was very limited, more so than ever before, as the process orientation had been pushed to the extreme within the entire hospital. While in the mid-1990s it mostly consisted of the patient-oriented day-to-day programme which organised the autonomous activities of various health professionals, in 2014/2015 it encompassed the entire hospital. Hence, the decreased locus of control in the work environment based on limited participation in decision-making led to frustration and dissatisfaction among Jo-care's people, because they could not maintain their professional standards and values in their clinical practice.

While in the 1990s the hospital's CEO and his partners were concerned with the creation of an organisational philosophy to positively influence the values, beliefs and practices of Jo-care's people, in 2014/2015 the new CEO and his six-member directorate relied on the structural as well as procedural implementation of the information technology-enhanced normative mechanisms to maintain centralised control. With this in mind, the expansion of people's participation in managerial decisions, the rise in the amount of accessible information and an increase in autonomous decision-making of Jo-care's people became insignificant.

Although the introduction of an organisational and human resource development programme had led to the involvement of Jo-care's people in somewhat self-managed project groups or quality circles, the CEO and his executive and senior managers were very aware of and banked on the human creative capital and its importance to the successful development of the hospital.

I was not at that seminar, and I am thinking to myself, you know what, I will never attend a seminar like that because I do not want to work on those things. But I saw the results, so I know if I put no work into it, then down the line I will definitely receive a warning letter from the senior medical consultant. (medical doctor)

The interview and observational data of both ethnographic visits suggest an increased use of the knowledge and experience of Jo-care's people, and considerable benefits arose from their involvement, both in monetary and innovative terms, without rewarding contributing employees. Another problem was that Jo-care's people had the impression that regardless of their proposals and suggestions, the desired outcomes of project groups were already set. As a consequence, they felt their knowledgeable input was not taken seriously.

The participative and educational programme had become firmly established in the day-to-day practice of Jo-care. Data from both ethnographic visits support the notion that Jo-care's people experienced fatigue and felt “worn down” by the increased expectations in the hospital's day-to-day practice. Today, contrary to the mid-1990s, Jo-care's people cannot withdraw from their involvement in working groups as their participation is an important part of the bureaucratic control mechanisms to develop streamlined hospital processes while maintaining high-quality patient care. During both ethnographic visits, employees clearly recognised their expected behaviour as outlined in Jo-care's rhetoric. Despite their conformity with the required work role, several of them used defence mechanisms and misbehaviours on a regular basis.

That's the way it is. They just take a close look at it to make sure the money keeps flowing. This surely does not mean the patients are cared for to a lesser extent, but especially this hair-splitting, how often did the patient drink or eat, how often, were his pants changed, etc. You are generous in the documentation. You suit it to fit. (staff nurse)

All the nurses were constantly searching for documentary solutions to amend uncompleted nursing tasks and make them appear as being accomplished, leading to frequent retrospective falsification of patient records. This kind of misbehaviour is a far cry from the professional ethos of nursing practice. Another form of misbehaviour was that charge nurses examined the final documentation after patient discharge and, if necessary, augmented patient records to the in-patient prospective payment system. Despite these forms of misbehaviour, nursing employees made attempts to gain control over their working world, time and space by using different collective strategies and forms of resistance.

### Physical and mental work intensification

Ethnographic insights from both visits indicate a strong endorsement of service orientation by the frontline nursing staff. Both in 1996/1997 and 2014/2015, Jo-care's people showed a strong commitment towards patient-orientation, healthcare quality and accountability, even prior to the implementation of the patient-oriented day-to-day programme. Yet, people's pre-commitment did not exclude them from managerial interventions to manage and mobilise them in pursuit of increased productivity and organisational efficiency, next to higher healthcare quality.

The reorganisation of professional healthcare actions and inter-professional interaction, communication and cooperation, labelled in 1996/1997 as “service-orientation towards people and people's needs” and termed “process orientation and lean management” in 2014/2015, were an attempt to link management objectives to policies and practices in the clinical labour process. There was evidence of “the manipulation of the context of nursing work combined with the continuation of traditional autonomy” ( Ackroyd and Bolton, 1999 , p. 383) in Jo-care. In 2014/2015, the manipulation of the context was enhanced and intensified by including clinical wards, departments of diagnostic and therapy as well as administrative areas within the hospital. By means of optimising the hospital's processes and inter-professional interfaces, Jo-care's CEO and his executive and senior managers wanted to guarantee the clinical workflow and eliminating the bottlenecks in the healthcare service line.

Well the aim is the age-old struggle between or with the framework, i.e. with the finance of healthcare. What is involved, then, is always furthering optimisation, so as to remain in an economically sound wake, which we really had permanently been able to do since [name of former CEO] took over in the early 1990s and, subsequently, to maintain the investment capability of our hospital, enabling further investment in new medical procedures, in suitably well trained personnel, and also in the architectural infrastructure. (CEO)

Many occupational groups find themselves increasingly under pressure, not only in terms of working time and effort but also in terms of their work autonomy. In 2014/2015, advanced technological innovations provided more sophisticated control mechanisms, with performance data being available in detail, and thus, to exert pressure on employees. Yet, just like in 1996/1997, Jo-care's people retained their professional control over their clinical work due to the “technical complexity” of their healthcare labour and the uncertain nature of the everyday hospital practice ( Dent, 1998 , p. 218).

And to me that is, the great success we have had over the last years in nursing because our charge nurses developed their own dynamic and have become considerably more independent, and no longer wait, that we make decisions up here at the CNE office. That is still not enough for me momentarily, well, I would like for them to take on a more active role in regards to autonomy. (CNE)

The findings in 1996/1997 and 2014/2015 with regard to physical and mental work intensification indicate that while the process orientation advanced service quality and labour productivity, it resulted in very high levels of physical activity and mental effort in caring for patients at a higher level of acuity. The continuing staff development was seen as an integral part of the managerial rhetoric, and Jo-care's people benefitted from the educational initiatives, such as acquiring skills which were also valued outside the hospital's day-to-day practice.

On the flipside, enhancement of employees' skills since 1996/1997 also meant the flexible deployment of nursing staff, greatly intensifying work stress and intellectual effort. Staff nurses were also required to work on a variety of ward floors within different medical disciplines. This strategy was supplemented by the multidisciplinary use of beds on the ward floors. As a result, staff development initiatives were a significant increase of pressure on the individual nurse. The outcomes of both ethnographic analyses suggest that Jo-care's people are working harder, contradicting the prescriptive findings of the institutional excellence advocates who promise the phenomenon of working smarter and not harder.

### Trust, commitment and control

A key theme of Jo-care's rhetoric in 1996/1997 was the creation of high trust work relations in order to reinforce and instil positive values and path-finding visions, which, in turn, generate enthusiasm, excitement and commitment amongst Jo-care's people. Using the tool Management by Walking About (MBWA), the social and the physical proximity between executive managers and Jo-care's people had improved, but it also led to feelings of animosity due to the obvious display of face-to-face control ( Beil-Hildebrand, 2006) . In 2014/2015, MBWA was still performed, but the frequency of these walkabouts was reduced to at most once a week, with the subjects of the MBWA along with its face-to-face control being foremost the people in charge of wards and departments of diagnostic and therapy and not on the general nursing staff.

I rarely come into contact with the CNEs, in the past they used to briefly stop by the ward at least once a week, that was usually at noon on Fridays, and would ask how the week had been and how things are running, but that has not happened lately at all. […] If some precarious issue arises, then they are around and tell us what is important, but otherwise, I hardly have any contact to them. (staff nurse)

Hence, it is not surprising that in 2014/2015, nursing staff did not perceive MBWA as a very important managerial strategy to enhance high trust relations. Normative and bureaucratic control was now exerted on Jo-care's people using the structurally implemented communication channels, such as the monthly meetings between the CNE and the charge nurses or the annual performance review. Further, middle management, that is, charge nurses, was now responsible to manage employees. Although the form of management changed from 1996/1997 to 2014/2015, Jo-care's people ended up being managed rather “closer” than “distant” ( Foucault, 1979 ).

The findings of the ethnographic revisit in 2014/2015 confirm the importance of organisational sub-cultures related to trustful working relationships. For a majority of Jo-care's people, the ward team was the most relevant reason to stay and work for this particular hospital. Charge nurses were engaged in building their own team-family on their ward and supported staff nurses not only practically but also offered psycho-emotional assistance. Concurrently, the CNE and his deputy tried to encourage the leadership competencies and abilities of their charge nurses because leadership styles do influence sub-cultures.

In the beginning I was a little shocked by the responsibility we were given. These stories about human resource responsibilities and this responsibility for the budget, I felt queasy the first time I heard the sum, for I have a personnel budget of over one point two million a year. That is insane! (charge nurse)

In 1996/1997, there was substantial heterogeneity in the attitudes of Jo-care's people, both within the nursing division and between management and staff. Whereas persuasive information and communication opportunities or promises of co-operative working arrangements had reinforced the loyalty of Jo-care's people, managerial prerogative demands lead to perceived unfairness, eroding active employee commitment and shared understanding of others. Due to the obvious domination of managerial demands in 2014/2015, the heterogeneous amalgam of the mid-1990s had faded.

The hospital's CEO and his top managers clearly adopted the position of “playing the bosses” instead of “being the trustful colleagues”. This led to a psycho-emotional relief on part of Jo-care's people enabling them to feel and act as ordinary employees by rationalising their misbehaviour and resistance as an employees' right in times of physical and mental work intensification. The adaptation of the nursing documentation was largely accepted amongst the ward teams because the workload accelerated due to a higher patient throughput, a shorter length of stay and an increase of documentation tasks. In 2014/2015, it was clear that Jo-care's CNE and his deputy were aware and had accepted employee misbehaviour.

### The structuring of people's subjectivity

In 1996/1997, there was considerable doubt that the managerial rhetoric had any impact on the level of service-orientation towards people and peoples' needs, but Jo-care's people nevertheless structured their work activity in terms of service-orientation. In 2014/2015, the CEO and his top managers did not call for service-orientation anymore since the different cross-departmental meeting structures served as interaction, communication and cooperation platforms; however, Jo-care's people still incorporated service-orientation and, due to their professional ethos, kept on living the professional values, beliefs and practices of health and nursing care. Hence, Jo-care's CEO could implicitly rely on these traditional forms of people's loyalty and professionalism. The ultimate goal in relation to managerial objectives was the provision of a more efficient, flexible and innovative healthcare service achieved through people's active commitment to Jo-care. In 1996/1997, this commitment was achieved by peoples' participation and normative re-education, whereas in 2014/2015, the rhetoric was influenced by the constant communication of accounting numbers and operating figures with the aim to instil managerial thinking into the employees.

Well, you have to present accounting numbers when you want something. Before it was done like this: Look, here, well, here is the problem. Difficult, but we need more personnel. You should not even bother showing up today if you do not have operating figures. (charge nurse)

In 1996/1997, Jo-care's people gave an impression of incorporating “distancing behaviour, cynicism, deep acting and resigned behavioural compliance” rather than acceptance and endorsement of the required values. In 2014/2015, this range of responses was still found in the day-to-day practice but was less related to normative strategies. Cynicism mainly referred to bad hospital management and focused on the uneven access to resources. In addition, fatigue also had set in among employees, which they presented as considerable scepticism, leading to the conclusion that no transformation of values and attitudes had been achieved. The professional autonomy of skilled clinical people from direct managerial control, the internal cohesion and their commitment to healthcare service helps explain some aspects of the employees' behaviour found in Jo-care's day-to-day practice in 1996/1997 as well as in 2014/2015. For instance, one charge nurse developed a particular strategy to order disposable healthcare material for infected wounds which often caused ordering issues. On a regular basis, he took repulsive photos of the infected wounds and sent them to the purchasing manager in Jo-care, who would be overwhelmed by the view and would then order the desired goods.

Jo-care's people responded in many different ways to the rhetoric-reality gap between managerial words and deeds, ranging from pretenders to performers ( Tonkens *et al.* , 2013 ). One of the most obvious actions may be people's speech, because it was noticeable how much spoken evidence indicated a personal awareness of managerial motives and the mismatch between the managerial rhetoric and the reality of its day-to-day practice. Although in 1996/1997, the rhetoric produced high levels of uncertainty, mistrust and involuntary economic activity, in 2014/2015, Jo-care's process orientation was well developed, embracing the entire hospital organisation. The introduction of innovative processing departments, such as the central occupancy service, led to streamlined patient care, for example, decreased average length of stay. The reconstitution of shared interests and enhanced accountability not only made people work smarter and harder, but it was also relatively easy to achieve, because Jo-care's people wanted to maintain their professional ethos and standards in their day-to-day practice.

If one of us is absent, then someone else fills in. […] You know that if you do not go into work, I am the last option, if I do not go into work, then the other colleagues will be staffed at a minimum and will have a terrible day, you always have a little bit of a guilty conscience if you do not go into work on short notice. (staff nurse)

There was a wide range of misbehaviour observed in Jo-care in 1996/1997 as well as in 2014/2015. Nursing staff not only attempted to appropriate time and space but also took hospital food and goods for personal ends with the knowledge of their superiors. While Jo-care's people were clearly reluctant to overtly express their displeasure about effort intensification and complex managerial control, they were able to maintain a good sense of freedom, because they had the potential to exert some individual control over their work environment.

In 1996/1997, the researcher assumed that people's feelings of hostility would increase in the hospital, and thereby the likelihood of continued and developing forms of resistance such as sabotage, pilferage or time wasting would increase as well. The findings in 2014/2015 confirmed this assumption because an open resistance had entered the discursive scenery as Jo-care's people realised that the rhetoric of empowerment and participation was often nothing more than hot air.

Well, I am slowly but surely becoming allergic to anyone who, so to speak, constantly tells me we are going to make a lucky strike with this digital dictation business and who knows what else, the paperless hospital and so on. So far, for ten years, this has been nothing but an excuse not to do anything at all because no one really wants to deal with it. (senior medical consultant)

The evidence suggests that although Jo-care's people maintained a sense of personal and professional autonomy and showed service-oriented loyalty towards the patient clientele and the hospital, they were far from reaching the level of active commitment the CEO and his managers seemed to regard as the ultimate success of normative control. In 2014/2015, it became obvious that the sub-cultures on the wards and departments of diagnostic and therapy were the important entities in the hospital where reciprocal commitment was lived and trustful work relations were a constant aspect of professional interactions ( Lok *et al.* , 2011 ).

### Managers and the culture burden

In 1996/1997 as well as in 2014/2015, the only people who were convinced by Jo-care's rhetoric were its own creators. During the first ethnographic visit, it appeared that their commitment to their work and to the objectives of Jo-care's rhetoric was much more developed and effective than that of their employees. Some of the executive managers, like the CNE, were very aware of the contradictions and pressures faced by the implementers of the rhetoric, and both of them had a great deal of sympathy for the people on the ward and departmental floors, which they felt was not always the case with Jo-care's CEO and his partners. However, the executive managers were expected to involve and educate Jo-care's people and give them the opportunity to be involved. Hence, these managers at the executive level carried the “culture burden” ( Marks *et al.* , 1997 , p. 479). In addition, the hospital's rhetoric might have been used as an important resource through which managers were able to control Jo-care's people and establish their own right to exist. In 2014/2015, however, the situation had changed. In 2012, a managerial employee of a private for-profit hospital chain became CEO of Jo-care. Thus, he did not carry any culture burden, but carried the burden of delivering black numbers and successfully positioning the hospital in the regional and national healthcare market:

We are on the right track, because when you take a look at the [ranking]-list we are listed at position 53 out of 2,000 hospitals in Germany. Ranked 53, and we are the eleventh best hospital in Bavaria. […] That is just sensational, and in 2012/2013 we were among the top ten hospitals in Germany regarding patient satisfaction, that means in third place, and those are truly outstanding results. (CEO)

The imposition of efficiency and performance measurements along the managerial rhetoric of the hospital's process-orientation has meant that professional healthcare and nursing work is currently considered just another commodity in human resource planning.

Additional changes concerned the composition of the directive management board. In 2014/2015, the six-member management board was accountable to the hospital's CEO and, in an indirect way, to his human resources within central services (quality management, hygiene, etc.). Yet, the culture burden had been transferred to senior and middle managerial levels, as the strategy of decentralising managerial activities included bureaucratic as well as normative control mechanisms. In other words, the responsible autonomy on behalf of senior and middle managers brought about not only more accounting responsibilities for the senior people in charge of medical disciplines, wards and departments of diagnostic and therapy, but also produced much higher pressure to build and maintain a stable sub-culture within their areas.

The exercise of the power of office is a matter of the CEO and his six-member management board. Even though external forces in the healthcare sector do exist, Jo-care's CEO and his managers continually emphasised the pressing need to increase service quality and improve efficiency in order to function somewhat better than the norm. The original management team was expanded in 2014/2015 by senior and middle managers. With this control came implicit threats to personal interests such as career prospects or income security.

It is plausible to claim that only a very small minority of managers – in effect the elite that “personifies capital” – occupy positions of comparative privilege ( Braverman, 1974 , p. 405) as they have a “direct managerial identity of interest and outlook” as the ecclesiastical authority of this hospital organisation ( Eldridge *et al.* , 1991 , p. 64). However, according to Anthony (1990 , p. 3), people involved in the subordination and control of others are most likely to be captured by culture change and to be isolated in the process because they are driven not only by political and economic motives but also by their personal aspiration to “superimpose the values and purposes” of “their organisation” upon their subordinates. The systems of bureaucratic, normative and technological control along with their consequences seem to disguise the healthcare reality in an environment that claims a major role in supporting not only the patient clientele but also the people who care for and nurse them.

## Final remarks and conclusion

This ethnographic revisit is about nursing professionals within one hospital organisation “who work far too hard for too few rewards and are constantly pressured” by Jo-care's CEO and his managers while making every attempt to support not only the patient clientele but also their fellow nurses ( Cooke, 2006 , p. 239). Its purpose was to explore and describe the rationale, method, building elements and impact of continuing corporate culture change initiatives in the same hospital organisation 17 years later. As the strong service attitude in relation to a professional ethos remains the ideological base of healthcare professions and as Jo-care's people are able to organise themselves as well as pursue a certain degree of autonomy, a second critical account of the mobilisation of corporate culture change is provided. This study confirms the results of the first ethnographic visit and supports accounts of effort intensification in nursing, while it also agrees with the more critical management literature which has highlighted the contradictions of the mobilisation of corporate cultures in the name of institutional excellence. Nursing employees were subjected to contradictory expectations and pressures – but so, too, were their executive, senior and middle managers. Key contradictions were processes of responsible autonomy which assumed to empower nursing staff, coinciding with a tightening of bureaucratic and normative control mechanisms, arbitrary implementations of managerial control and a management style which confused elements of both high and low trust in an unstable hospital world. Nonetheless, Jo-care's CEO was also constrained to act as a capitalist due to his duties to purchase nursing labour power and harness its productive efforts, knowledge and creativity. This takes on a distinct form in that his superior is the ecclesiastical hospital authority rather than the free market. The imposition of efficiency and performance measurements along with the managerial rhetoric of the hospital's process-orientation has meant that professional nursing work is currently considered just another commodity in human resource planning. Thus, the labour process of professional nurses is now managed and controlled as if it were for-profit, and distinguishing the top management of a religious hospital from those of commercial organisations is no longer an easy task. What are the implications for clinical and managerial practitioners? The recommendations are to (1) develop managerial practitioners who are capable of managing change combined with the professional autonomy of clinical practitioners, (2) take care to practise what you preach in clinical and managerial reality, as commitment, consent, compliance and difference of opinion are signs of a healthy corporate culture and (3) consider the implications between social structures and human actions with different work behaviours on different levels involved.
